# Dual Oxidase Maturation factor 1 (DUOXA1) overexpression increases reactive oxygen species production and inhibits murine muscle satellite cell differentiation

**DOI:** 10.1186/1478-811X-12-5

**Published:** 2014-01-11

**Authors:** Shelley DE Sandiford, Karen AM Kennedy, Xiaojun Xie, J Geoffrey Pickering, Shawn SC Li

**Affiliations:** 1 Siebens-Drake Research Institute, 1400 Western Road, London, Ontario N6G 2 V4, Canada; 2 Department of Biochemistry and the Siebens-Drake Medical Research Institute, Schulich School of Medicine and Dentistry, Western University, London, Ontario N6A 5C1, Canada

**Keywords:** Muscle satellite cell, Stem cell, Myogenesis, Reactive oxygen species

## Abstract

**Background:**

Dual oxidase maturation factor 1 (DUOXA1) has been associated with the maturation of the reactive oxygen species (ROS) producing enzyme, dual oxidase 1 (DUOX1) in the adult thyroid. However, ROS have also been implicated in the development of several tissues. We found that activated muscle satellite cells and primary myoblasts isolated from mice express robust levels of DUOXA1 and that its levels are altered as cells differentiate.

**Results:**

To determine whether DUOXA1 levels affect muscle differentiation, we used an adenoviral construct (pCMV5-DUOXA1-GFP) to drive constitutive overexpression of this protein in primary myoblasts. High levels of DUOXA1 throughout myogenesis resulted in enhanced H_2_O_2_ production, fusion defects, reduced expression of early (myogenin) and late (myosin heavy chain) markers of differentiation, and elevated levels of apoptosis compared to control cells infected with an empty adenoviral vector (pCMV5-GFP). DUOXA1 knockdown (using a DUOXA1 shRNA construct) resulted in enhanced differentiation compared to cells subjected to a control shRNA, and subjecting DUOXA1 overexpressing cells to siRNAs targeting DUOX1 or apoptosis signal-regulating kinase 1 (ASK1) rescued the phenotype.

**Conclusions:**

This study represents the first to demonstrate the importance of DUOXA1 in skeletal muscle myoblasts and that DUOXA1 overexpression in muscle stem cells induces apoptosis and inhibits differentiation through DUOX1 and ASK1.

## Background

The process of myogenesis is often studied using activated satellite cells. These muscle stem cells, located between the plasma membrane and the basal lamina, form the basis for effective muscle regeneration [1]. Under appropriate stimuli, these normally quiescent cells enter back into the cell cycle, and undergo several rounds of proliferation. Myoblast progression towards mature muscle is initiated by permanent cell cycle exit. These cells, now called myocytes, line up and fuse with neighboring cells to produce a single-membrane structure housing potentially hundreds of nuclei. The process of myogenesis is dependent upon the expression of the Myogenic Regulatory Fators (MRFs) that include Myf5, MyoD, myogenin and MRF4 [2]. Both MyoD and Myf5 are expressed in proliferative myoblasts and Myf5 is downregulated as cells progress through myogenesis. Once the cells exit the cell cycle, myogenin and MRF4 are expressed. MRF4 can also act upstream of Myf5 and MyoD [3].

Although there appears to be a certain degree of redundancy between the MRFs, data from knockout studies suggest unique roles for these transcription factors. The majority of myoblasts follow this rather predictable pattern of myogenesis and, in mature muscle, most of the nuclei are terminally differentiated. However, the process of myogenesis is also characterized by a small percentage of cells that escape differentiation, maintain Pax7 expression, downregulate MyoD, and return to quiescence [4]. These Pax7^+^/MyoD^-^ cells are thought to maintain a small pool of muscle stem cells, from which future proliferative myoblasts may be derived. Cells that escape differentiation and that fail to return to quiescence undergo apoptosis [5,6]. Indeed, apoptosis is normally regarded as a natural part of differentiation, and identifying factors involved in cell cycle control and survival undoubtedly play an important role in our general understanding of myogenesis and in the etiology of many muscle degenerative diseases.

Previously, our lab characterized a protein termed Numb Interacting Protein (NIP), an interactor of the cell fate determinant, Numb, in *Drosophila Melanogastor*[7]. Mammals have two isoforms of NIP (NIP1 and NIP2), but the role of mammalian *NIP* genes in Numb function has not been demonstrated. Subsequently, others identified *NIP1* and *NIP2* as genes arranged in head-to-head orientation with dual oxidases (*DUOX1* and *DUOX2*) [8]. Specifically, the protein products of these genes, renamed DUOXA1 and DUOXA2 respectively, were shown to be important for the maturation of DUOX1 and DUOX2 and, ultimately, the production of H_2_O_2_ and thyroid hormone. Dual oxidases belong to the nicotinamide adenine dinucleotide phosphate (NADPH) oxidase (Nox) family of enzymes responsible for the generation of reactive oxygen species (ROS) in a variety of cell types [9-12]. The family is made up of Nox1 through Nox5 and DUOX1 and DUOX2. While some family members require additional subunits (p47^phox^ and p67^phox^) for proper function, DUOX1 and DUOX2 have no such requirement [13,14]. Instead, these two Ca^2+^-dependent Nox members rely on DUOXA1 and DUOXA2 for their maturation and/or translocation to the plasma membrane for their activation. Research has demonstrated that DUOXA1 and DUOXA2 form heterodimeric complexes with their respective dual oxidases [15,16] and, in their absence, DUOX enzymes remain internalized in the endoplasmic reticulum and H_2_O_2_ is not produced. Interestingly, relatively few adult tissues have been demonstrated to express DUOXA1. Grasberger and Refetoff [8] confirmed high levels of this protein in a limited number of tissues including the thyroid, lung and salivary glands. Indeed, most of the studies on DUOXA1 and DUOXA2 revolve around their function in the thyroid and hormone biosynthesis and, not surprisingly, natural mutations in the *DUOXA2* gene have been linked to hypothyroidism [17,18]. However, the presence of DUOX and DUOXA in primitive organisms (lacking a thyroid gland), suggests roles that extend beyond thyroid hormone biosynthesis [19]. Others have suggested that DUOX1 in lung epithelia may play a role in host defence [20], and silencing of *DUOX1, DUOX2* and their respective maturation factors has been demonstrated in lung cancer cells [21]. Since 2006, DUOXA1 has been studied extensively as a mediator of DUOX1 activity. However, studies into the potential roles for DUOXA1 in other tissues and during development are lacking.

We have determined that *DUOXA1* mRNA levels are altered throughout embryogenesis and that levels are elevated as early as embryonic (E) day seven (E7) in the developing mouse [22]. The early expression pattern of DUOXA1 (before the development of many organs) suggests that it may play important roles in embryogenesis. Here we report, for the first time, that DUOXA1 (and its corresponding dual oxidase, DUOX1) is expressed in murine muscle satellite cells and throughout myogenesis. Overexpression of DUOXA1 is associated with elevated levels of H_2_O_2_ and inhibition of differentiation through increased apoptosis in a DUOX1-dependent manner. We further show that a common regulator of apoptosis, apoptosis signal-regulating kinase 1 (ASK1), is a downstream target of DUOXA1-mediated H_2_O_2_ production, and that knockdown of either DUOX1 or ASK1 rescues the DUOXA1 overexpression phenotype.

## Results

### Newly activated satellite cells and primary myoblasts express DUOXA1

**Figure 1:**
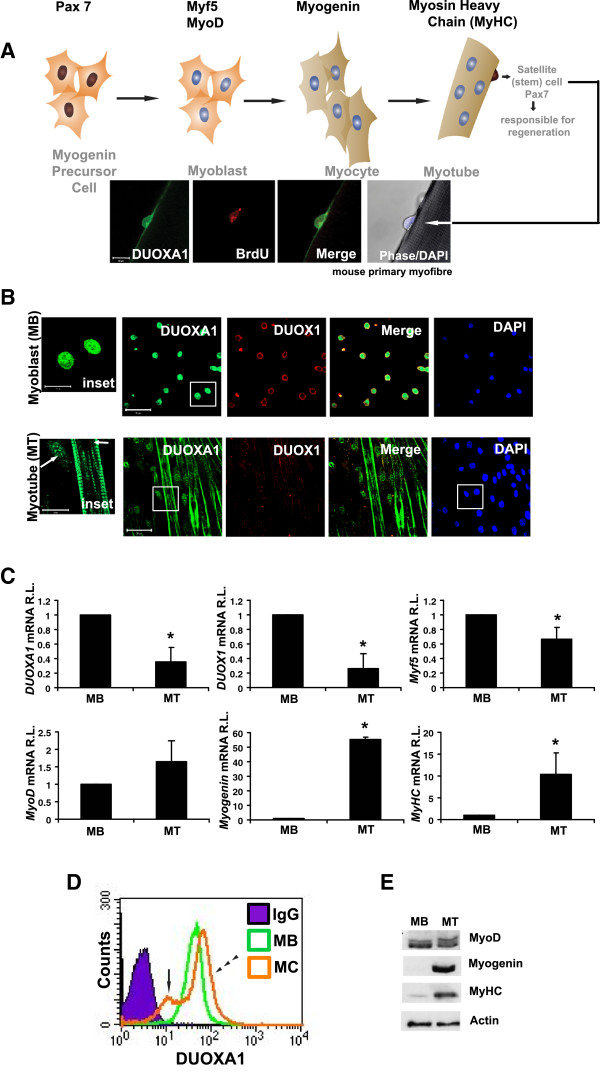
**Newly activated satellite cells and primary myoblasts express DUOXA1. (A)** Scheme of myogenesis indicating common markers for precursor cells (Pax7), myoblast commitment (Myf5, MyoD), early differentiation (myogenin) and late differentiation (Myosin heavy chain - MyHC). Myofibres were isolated and incubated with 10 μM bromodeoxyuridine (BrdU). Samples were cultured for 24 hours, upon which time they were harvested, fixed in 2% paraformaldehyde, and immunostained for DUOXA1 (green) and BrdU (red). Results show evidence of activated DUOXA1^+^/BrdU^+^ satellite cells. Scale bar: 20 μm. **(B)** Myofibre and primary myoblast cultures obtained from the extensor digitorum longus muscles of adult mice demonstrate DUOXA1 expression in newly-activated satellite cells, primary myoblasts and differentiated myotubes. Primary myoblasts which have migrated away from the parent fibre also show extensive cytoplasmic and nuclear staining of DUOXA1 (green) in cells that co-express DUOX1 (red). The nuclei of differentiated myotubes are generally devoid of DUOXA1. White arrows indicate alterations in the localization of DUOXA1 in single cells compared to that in fused myotubes. Counterstaining with DAPI is provided as a nuclear marker. Scale bars: 50 μm. Inset scale bars: 20 μm. **(C)** The expression of *DUOXA1* and *DUOX1* (along with *Myf5, MyoD, myogenin & MyHC*) in myoblast (MB) and myotube (MT) samples was analyzed by quantitative reverse transcription (qRT)-PCR. The levels of mRNA were normalized to *GAPDH* and presented as values relative to expression at day 0 (d0). **(D)** DUOXA1 levels in MB and myocyte samples (MC - which had not undergone fusion) were also analyzed by flow cytometry. The normal arrow and broken arrow heads in MC samples indicate populations of cells that have either downregulated or upregulated DUOXA1 expression respectively. **(E)** Markers of myogenesis were also analyzed by Western blot. * Significantly different from d0 (P < 0.05). mRNA R.L. indicates mRNA relative level.

To determine whether muscle satellite cells express DUOXA1, myofibre cultures derived from mouse extensor digitorum muscle were examined by immunofluorescent microscopy. Robust DUOXA1 expression was detected at 24 hrs of culture in cells that had entered back into the cell cycle (as demonstrated by positive BrdU staining (Figure 1A). In order to characterize the function of DUOXA1, we generated an anti-DUOXA1 antibody against the C-terminal portion of the mouse DUOXA1 protein. The specificity of the antibody was verified by overexpressing full length DUOXA1 in 293T cells, and by immunostaining performed on primary myoblasts in the absence or presence of the antigenic peptide (Additional file 1: Figure S1A-D). The antibody was also verified using the immortalized C2C12 myoblast cell line (Additional file 1: Figure S1E).

We were also interested in knowing whether DUOXA1 expression was maintained in primary myoblasts that had migrated from the parent fibre. Primary myoblasts were derived from myofibre cultures, and culture purity was determined to be > 95% using the myoblast marker, desmin (data not shown). Immunostaining performed on proliferative myoblast (MB) and differentiated myotube (MT) samples suggest that DUOXA1 is present in the nucleus and cytoplasm of dividing myoblasts, and restricted to the cytoplasm of fused myotubes (Figure 1B).

### Dynamic DUOXA1 expression during myogenesis

We next examined the temporal expression pattern of *DUOXA1* as cells undergo differentiation. Proliferative primary myoblasts were either maintained in growth medium (GM), or allowed to differentiate for four days in differentiation medium (DM). Quantitative reverse transcription (qRT)-PCR suggests that DUOXA1 mRNA levels are altered as cells differentiate (Figure 1C and E). Due to differences in DUOXA1 localization between proliferating and differentiating cells, we decided to use flow cytometry as a means of further characterization. Flow cytometry performed on proliferative MB and on differentiating myocytes (MC – harvested before the process of fusion) suggests that separate populations of DUOXA1 emerge (Figure 1D). Taken together, these results suggest that DUOXA1 is a highly dynamic protein whose levels and localization depend on whether samples are dividing or differentiating.

### DUOXA1 overexpression inhibits myogenesis

**Figure 2:**
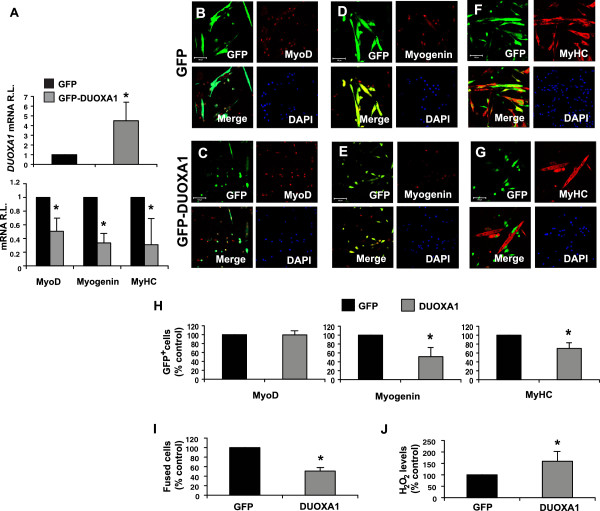
**DUOXA1 overexpression in primary myoblasts inhibits differentiation.** Primary myoblasts were infected with adenoviral vectors containing pCMV5-GFP (GFP), or pCMV5-DUOXA1-GFP (GFP-DUOXA1). **(A)** Cells were subjected to adenoviral constructs and, 24 hours later, growth medium (GM) was replaced with differentiation medium (DM - designated as day 0). mRNA was harvested 24 hours after the initiation of differentiation (day 1). Quantitative RT-PCR was performed on representative samples from DUOXA1 overexpressing cells and GFP controls. Data was normalized to *GAPDH* and GFP-DUOXA1 values are presented relative to GFP control cells. *MyoD*, *myogenin* and *MyHC* mRNA levels are all significantly reduced in samples overexpressing DUOXA1. **(B-H)** Immunostaining on samples harvested on day 2 suggests the number of GFP^+^/myogenin^+^ and GFP^+^/MyHC^+^ cells are clearly reduced in DUOXA1 overexpressing cells **(E, G, H)** compared to GFP controls **(D, F, H)**. Numbers of GFP^+^/MyoD^+^ cells are not different between samples **(B, C, H)**. Counterstaining with DAPI is provided as a nuclear marker. Scale bars: 100 μm. **(H)** Graphical representation of the data shown in **B**-**G**. GFP-DUOXA1 counts are represented relative to GFP controls. **(I)** The ability of cells overexpressing DUOXA1 to fuse is also impaired compared to GFP control cells collected at day 2. **(J)** In order to assess the amount of H_2_O_2_ released from the cells, the amplex red reagent was used to demonstrate that DUOXA1 overexpression results in significant increases in H_2_O_2_ in the culture medium. Levels of H_2_O_2_ were corrected using cell counts and the amount of H_2_O_2_ in DUOXA1 overexpressing cells are expressed relative to GFP controls. Values represent means from myoblasts isolated from 3–5 mice ± SE. * Significantly different from GFP control (P < 0.05).

In order to determine whether altering the levels of DUOXA1 might have an impact on myogenesis, we created an adenoviral vector containing full-length mouse DUOXA1 (pCMV5-DUOXA1-GFP). Virus containing the empty vector (pCMV5-GFP) was used as the corresponding control. Cells were infected, and induced to differentiate 24 hrs later (designated as day 0). The efficiency of adenoviral infection in primary myoblasts was calculated to be 70-80% (data not shown). Quantitative RT-PCR at day 1 suggests that DUOXA1 overexpression reduced markers of early (*myogenin*) and late (*myosin heavy chain – MyHC*) differentiation by 66.4% and 69.1%, respectively (P < 0.05, Figure 2A). Similarly, *MyoD* mRNA was also reduced by 49.5% in cells overexpressing DUOXA1 (P < 0.05, Figure 2A). Confocal immunofluorescence was performed on samples harvested at day 2 of differentiation. Although the numbers of MyoD^+^(red)/GFP^+^(green) cells were not significantly different between samples (Figure 2B, C and H), there was a 48.4% reduction in the number of myogenin^+^(red)/GFP^+^(green) cells in DUOXA1 overexpressing samples compared to GFP cells (P < 0.05, Figure 2D, E and H). Similarly, immunostaining with an antibody against MyHC revealed a 29.8% decrease in the number of MyHC^+^(red)/GFP^+^(green) cells infected with DUOXA1, compared to GFP control samples (P < 0.05, Figure 2F, G and H). The ability of cells to fuse was also hindered in DUOXA1 overexpressing cells (P < 0.05, Figure 2I).

### DUOXA1 overexpression results in an increase in H_2_O_2_ production

It has previously been determined that the translocation and maturation of DUOX1, and the subsequent production of H_2_O_2_, is dependent on the expression of DUOXA1 [8]. Having established the effects of DUOXA1 overexpression on myogenic differentiation, we questioned whether overexpression also resulted in alterations in the production of H_2_O_2_. Previous reports of DUOX1 expression in myoblasts had not been demonstrated, but we determined by immunostaining that DUOX1 was located primarily at the plasma membrane in these cells. We utilized an amplex red reagent to establish that DUOXA1 overexpressing cells indeed released more H_2_O_2_ into the surrounding medium than did GFP control cells. Overexpression resulted in a 59.3% increase in the levels of H_2_O_2_ (P < 0.05, Figure 2J). Hence, DUOXA1 overexpression resulted in elevated levels of H_2_O_2_, compromised fusion and inhibited differentiation.

### DUOXA1 overexpression elevates apoptosis signal-regulating kinase (ASK) 1 expression and induces apoptosis in primary myoblasts undergoing differentiation

**Figure 3:**
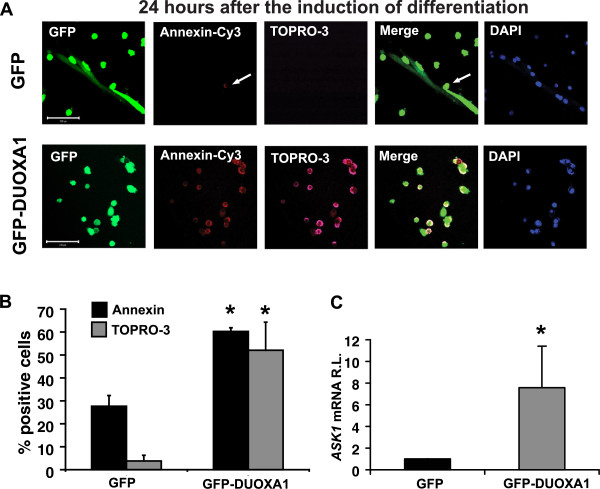
**DUOXA1 overexpression elevates apoptosis signal-regulating kinase (ASK) 1 levels and induces apoptosis. (A-B)** Cells were infected with pCMV5-GFP (GFP), or pCMV5-DUOXA1-GFP (GFP-DUOXA1). Twenty-four hours after infection, growth medium was replaced with differentiation medium and cells were harvested 24 hr later (day 1). **(A)** Apoptosis was measured by labelling samples with Annexin V-Cy3 (red) and propridium iodide (TOPRO-3 - magenta). Cells overexpressing DUOXA1 undergo massive amounts of apoptosis, as evidenced by cells undergoing early (Annexin V^+^/TOPRO-3^-^) and late (Annexin V^+^/TOPRO-3^+^) apoptosis. Scale bars: 100 μm. **(B)** Graphical representation of data presented in **(A)**. Counterstaining with DAPI is provided as a nuclear marker. **(C)** Levels of *ASK1* in GFP and GFP-DUOXA1 samples were analyzed by qRT-PCR. Samples were normalized to *GAPDH* and GFP-DUOXA1 values are displayed relative to GFP controls. Data suggest upregulation of *ASK1* in samples overexpressing DUOXA1. * Significantly different from GFP control (P < 0.05).

Within 48 hours of differentiation, DUOXA1 overexpressing cells appeared to be dying. Therefore, we assessed whether overexpression resulted in enhanced apoptosis during differentiation. We used AnnexinV-Cy3 and propridium iodide (TOPRO-3) to determine that, by day 1 of differentiation, DUOXA1 overexpression resulted in more than double the number of Annexin positive cells and over ten times the number of TOPRO-3 positive cells compared to GFP controls (P < 0.05, Figure 3A and B), indicating significant increases in the number of cells undergoing early (Annexin positive) and late (Annexin, TOPRO-3 double positive) apoptosis. We next sought to determine whether enhanced apoptosis was associated with elevated levels of ASK1, a common mediator of apoptosis. Quantitative RT-PCR indicated that DUOXA1 overexpressing samples had significantly elevated levels of *ASK1* mRNA by five hours post infection (P < 0.05, Figure 3C).

### DUOXA1 knockdown results in enhanced differentiation

**Figure 4:**
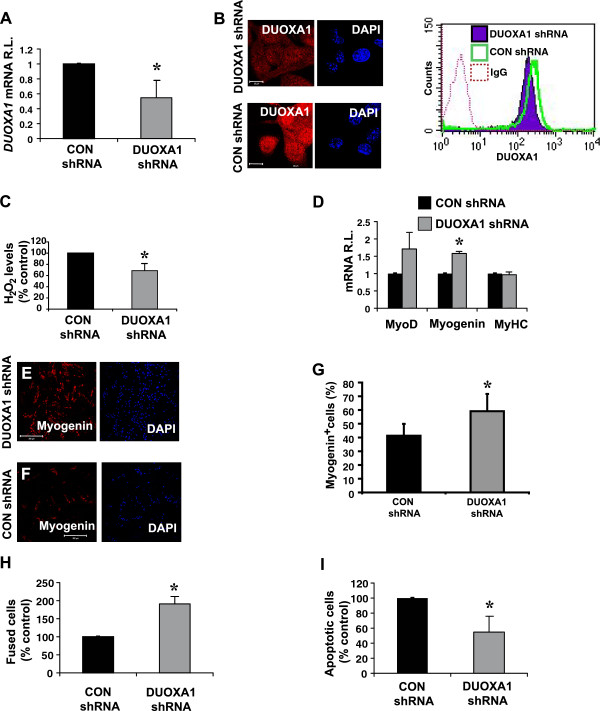
**DUOXA1 knockdown decreases H**_**2**_**O**_**2**_ **production and enhances differentiation.** Primary myoblasts were subjected to nucleofection using shRNA constructs targeting DUOXA1 (DUOXA1 shRNA) or luciferase (CON shRNA). Twenty-four hours later, growth medium was replaced with differentiation medium, and samples were harvested 48 hours after the initiation of differentiation (day 2). **(A)** Quantitative RT-PCR was performed on representative samples subjected to DUOXA1 shRNA and CON shRNA. Data was normalized to *GAPDH* and DUOXA1 shRNA values are presented relative to CON shRNA cells. **(B)** Confocal immunofluorescence and flow cytometry were used to demonstrate the ability of the DUOXA1 shRNA constructs to reduce DUOXA1 protein levels. Scale bars: 20 μm. **(C)** Amplex red was used to illustrate that DUOXA1 knockdown reduces the amount of H_2_O_2_ released by the cells. Levels of H_2_O_2_ were corrected using cell counts, and the amount of H_2_O_2_ produced by DUOXA1 shRNA cells are expressed relative to CON shRNA samples. **(D)** Quantitative RT-PCR was used to confirm significantly higher levels of myogenin, but not MyoD or MyHC, in DUOXA1 shRNA samples. **(E-G)** Immunostaining on differentiating samples suggests that the number of Myogenin^+^ cells is higher in DUOXA1 shRNA **(E)** samples than in controls **(F)**. Scale bars: 200 μm. **(G)** Graphical representation of some of the data in **E** &**F**. The number of MyoD^+^ cells was not different between groups. **(H)** At day 2, enhanced fusion was associated with DUOXA1 knockdown. **(I)** Annexin staining on samples subjected to DUOXA1 shRNA or CON shRNA suggest that DUOXA1 knockdown results in significantly reduced apoptosis. Values represent means for myoblasts isolated from 3–5 mice SE. * Significantly different from CON shRNA (P < 0.05).

In order to further characterize a role for DUOXA1 in myogenesis, we used shRNA constructs targeting two separate regions of the DUOXA1 gene (DUOXA1 shRNA). A construct targeting luciferase was used as the corresponding control (CON shRNA). Data from one shRNA construct is depicted in Figure 4. DNA was introduced into the cells by nucleofection and, 24 hrs later, GM was replaced by DM. Samples were harvested on day 2. We demonstrated that DUOXA1 knockdown reduced *DUOXA1* mRNA and protein using qRT-PCR, immunofluorescence and flow cytometry (Figure 4A and B). The amount of H_2_O_2_ released from the cells was also reduced by 31% (P < 0.05, Figure 4C). Quantitative RT-PCR demonstrated that, while MyoD and MyHC were not differentially altered by DUOXA1 knockdown, there was a 58.7% increase in *myogenin* mRNA (P < 0.05, Figure 4D). Similarly, the number of Myogenin^+^ cells was increased upon DUOXA1 knockdown (P < 0.05, Figure 4E-G). The number of MyoD^+^ cells was not different between groups. Additionally, DUOXA1 knockdown resulted in a 91% increase in fusion (P < 0.05, Figure 4H), and led to a 45% decrease in the number of cells undergoing apoptosis, as measured by AnnexinV staining (P < 0.05, Figure 4I). Taken together, these data suggest that DUOXA1 knockdown reduces the levels of H_2_O_2_, enhances early markers of differentiation and the ability of cells to fuse.

### The phenotype associated with DUOXA1 overexpression can be alleviated by DUOX1 or ASK1 depletion

**Figure 5:**
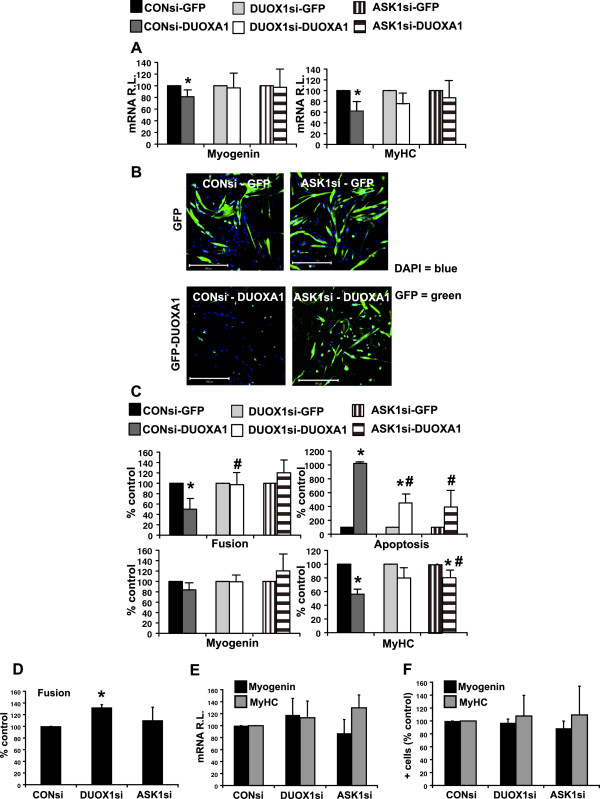
**DUOXA1 inhibits myogenesis through a mechanism involving DUOX1 and ASK1.** Small interfering RNAs targeting DUOX1 (DUOX1 siRNA), ASK1 (ASK1 siRNA) or a scrambled control (CON siRNA) were introduced into myoblasts using nucleofection. After 24 hrs, samples were subjected to adenoviral vectors containing GFP-DUOXA1 (DUOXA1) or GFP. **(A)** Quantitative RT-PCR was performed on samples harvested on day 1. Data was normalized to *GAPDH* and DUOXA1 values are presented relative to corresponding GFP controls. Reductions in the levels of *myogenin* and *MyHC* mRNA associated with DUOXA1 overexpression are alleviated upon knockdown of DUOX1 or ASK1. **(B)** Images demonstrating the ability of ASK1 siRNA to rescue the DUOXA1 phenotype. GFP is visualized in samples. Scale bars: 500 μm. **(C)** Graphical representation of cell counts. Confocal immunofluorescence was performed on samples harvested at day 1, and the number of cells expressing Myogenin and MyHC were counted. The number of MyHC^+^ cells was reduced in samples subjected to CON siRNA and DUOXA1 overexperssion compared to CON siRNA and GFP controls. This was alleviated by ASK1 knockdown. Counts are represented relative to their corresponding controls (e.g. DUOX1si-DUOXA1 relative to DUOX1si-GFP). The large number of Annexin-V^+^ cells witnessed upon DUOXA1 overexpression and defects in cell fusion can be reversed with siRNAs targeting DUOX1 and/or ASK1. **(D-F)** To determine whether DUOX1 or ASK1 knockdown alone would have an effect on differentiation, we subjected samples to DUOX1 siRNA or ASK1 siRNA and a suitable control (CON siRNA). DUOX1 knockdown enhances fusion **(D)**, but not Myogenin or MyHC mRNA **(E)** or protein levels **(F)**. ASK1 knockdown had no effect on differentiation. *Significantly different from samples infected with the corresponding GFP control (P < 0.05. **#** Significantly different from samples nucleofected with a scrambled control (CON siRNA) and infected with a DUOXA1 construct (CONsi-DUOXA1, P < 0.05).

**Figure 6:**
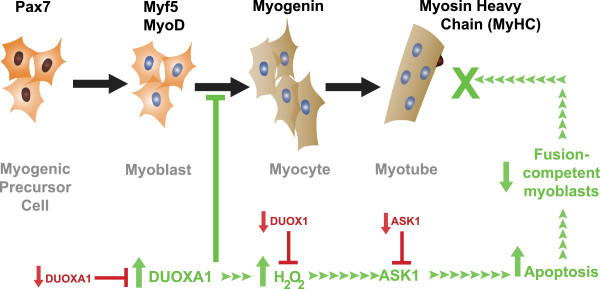
**A model for the effects of DUOXA1 on muscle differentiation.** The proposed model suggests that elevated levels of DUOXA1 act downstream of MyoD to initiate massive cell death and impair differentiation. DUOXA1 knockdown enhances differentiation. Samples subjected to siRNAs targeting DUOX1 or ASK1 rescues the overexpression phenotype. The effects of DUOXA1 overexpression and knockdown/rescue are presented in green and red, respectively, and suggest that DUOXA1-mediated apoptosis inhibits differentiation through a mechanism involving DUOX1 and ASK1.

The association between DUOXA1 and DUOX1 in other cell types is well established [8,15,16,22]. In order to determine whether the DUOXA1 phenotype was DUOX1 and/or ASK1-dependent, we subjected primary myoblasts to siRNAs targeting DUOX1, ASK1 or a scrambled control by nucleofection. Twenty-four hours after nucleofection, samples were infected with adenoviral constructs containing GFP-DUOXA1 or a GFP control and, 24 hours later, differentiation was induced. Cells were harvested after 24 hours of differentiation. Samples subjected to both scrambled control siRNA and DUOXA1 overexpression demonstrated an 18.8% decrease in *myogenin* mRNA and a 37.9% decrease in *MyHC* mRNA compared to control cells (P < 0.05, Figure 5A). Reductions in these two markers were alleviated by either DUOX1 knockdown or ASK1 knockdown. We used confocal microscopy and cell counts to determine that scrambled control siRNA cells overexpressing DUOXA1 experienced a 49.9% reduction in fusion (P < 0.05) which was reversed with either DUOX1 siRNA or ASK1 siRNA (Figure 5B and C). Similarly, the 43.8% reduction in MyHC witnessed with DUOXA1 overexpression (P < 0.05) was also alleviated upon knockdown of DUOX1 or ASK1 (Figure 5C). Levels of apoptosis common to DUOXA1 overexpression were also significantly lowered when these cells were subjected to DUOX1 or ASK1 depletion (P < 0.05, Figure 5C). Although there was a similar trend for myogenin, levels of this marker were not significant. In order to determine whether DUOX1 and/or ASK1 knockdown altered the ability of the cells to differentiate, we subjected samples to either DUOX1 siRNA or ASK1 siRNA and CON siRNA. We determined that ASK1 knockdown on its own had no effect on differentiation, while DUOX1 knockdown increased the ability of the cells to fuse, but had no effect on the expression of Myogenin or MyHC (Figure 5D-F). Based on these findings, we propose a model whereby DUOXA1 overexpression hinders differentiation and initiates apoptosis through mechanisms involving DUOX1 and ASK1 (Figure 6).

## Discussion

This report represents the first study to demonstrate the presence of a DUOX1-DUOXA1 system in activated satellite cells and primary myoblasts, and suggests an important role for DUOXA1 in normal myoblast function and differentiation. Our data imply that DUOXA1 levels and localization are altered as myoblasts differentiate, and that overexpression results in increased H_2_O_2_ production, apoptosis and defective differentiation. In agreement with our previous findings [22], we demonstrate that overexpression of DUOXA1 (and not the dual oxidase itself) can enhance H_2_O_2_ production in cells that already express DUOX1. The observation that endogenous DUOXA1 levels and localization change as cells differentiate is an interesting one. Flow cytometry data suggests that differentiation stimulates the emergence of two populations of cells with respect to DUOXA1 levels. The significance of these separate populations remains unclear. This pattern has been identified in other types of differentiating cells (data not shown) and suggests a level of caution be applied when analyzing DUOXA1 levels solely by Western blot.

The observation that adult skeletal muscle produces low amounts of ROS under resting conditions is well established, as is the importance of ROS in force development [23] and during myocyte disruption [24]. However, a potential role for endogenous ROS in myogenesis is poorly understood. Reactive oxygen species (ROS) are known to be important for the differentiation of cardiac [25-29], smooth muscle [30,31] and neuronal cells [22,32,33]. In skeletal muscle, it has been demonstrated that differentiation is naturally associated with elevated levels of ROS [34,35] and, similar to other tissues, there are reports suggesting that a rise in ROS is necessary to support differentiation and fusion [34-36]. Nox family members Nox1, Nox2 and Nox4 have been described in skeletal muscle and in myoblasts [19,35]. The ability of Nox proteins to mediate differentiation appears to be linked to ROS production, and the emerging picture is that proper control of development is tightly linked to ROS levels. Piao et al. [35] used siRNA against Nox1 and Nox2, and a range of inhibitors to determine that both knockdown of Nox2 and the use of ROS scavengers inhibit myogenesis. Even though alterations in myoblast DUOXA1 levels produce an opposite phenotype to that observed for Nox2, it is interesting to note that the characterization of DUOXA1 and DUOX1 in myoblasts represents the fourth Nox system to be described in these cells. Differences in temporal expression during differentiation, and resulting phenotypes from their knockdown or overexpression suggest that these enzymes may be activated by different stimuli, that they may signal through different pathways, and that they are likely not fully redundant.

It should also be noted that the immortalized C2C12 myoblast cell line is the model of choice in many investigations. Work in our lab suggests that C2C12 cells may be considerably more resistant to elevations in ROS levels than are primary myoblasts. Others have reported using mM levels of H_2_O_2_ to disrupt myogenesis. Although the exact level of H_2_O_2_ needed to induce catastrophic damage remains unclear, investigations confirming links between ROS and apoptosis in C2C12 cells typically use 0.5 mM to 4 mM H_2_O_2_[37-39]. Our preliminary data suggest that myogenesis can be inhibited using as little as 1–10 μM H_2_O_2_ in primary myoblasts (S.D.E.S, unpublished data), with the ability of the cells to fuse being particularly susceptible. We thus decided to focus our studies on primary myoblasts since we assumed the data would be more relevant than that derived from immortalized cells. However, one of the challenges of working with primary cells is the small sample sizes. Since many of the conditions applied in this study also resulted in cell death, we made the decision to focus primarily on cell counts, qRT-PCR and, where applicable, flow cytometry. Immunoblotting was not possible under these conditions. However, the data clearly demonstrate that high levels of DUOXA1 are detrimental to myogenesis and that its levels need to be strictly controlled. Future studies incorporating mouse and human primary cell models should begin to provide a clearer picture of the overall sensitivity of myoblasts to ROS and to provide a better understanding of how the Nox family of enzymes work to promote and inhibit myogenesis.

Proper skeletal muscle differentiation is dependent upon adequate pools of fusion-competent myoblasts. Apoptosis naturally occurs during differentiation, and there is some evidence to suggest that mediators of cell death are, in fact, required to initiate differentiation [6,40]. However, there appears to be some discrepancy between appropriate (and expected) levels of death associated with normal differentiation, and exaggerated levels of apoptosis resulting in severe reductions in cell numbers and hindered development. There are several reports indicating that controlling the level of apoptosis that occurs during differentiation may be therapeutically useful for a variety of degenerative diseases and aging [41-46].

Our results indicate that DUOXA1 overexpression can initiate the process of apoptosis through DUOX1 and ASK1. In our rescue experiments, DUOXA1 overexpression resulted in decreases in *Myogenin* mRNA but not protein. In other experiments (where levels of mRNA protein coincide with each other) cells were harvested after two days of differentiation. In our rescue experiments, samples were harvested after a single day of differentiation. This is due to the fact that the primary cells had been subjected to both adenovirus and nucleofection. Nucleofection is a very efficient method of gene transfer in primary myoblasts, but it also results in a small amount of toxicity. Since detectable differences in mRNA will always precede alterations in the level of protein, this earlier time point may have compromised our ability to detect larger differences in some of our parameters.

We discovered that ASK1 knockdown had no effect on differentiation. However, the observation that DUOX1 knockdown enhances the ability of the cells to fuse coincides with DUOXA1 data. It is curious that DUOX1 knockdown was not as effective as DUOXA1 at altering levels of Myogenin protein or RNA levels. While our data still suggests a connection between DUOXA1 and DUOX1 in the production of ROS and cell death in primary myoblasts, it is possible that DUOXA1 also has some DUOX1-independent role(s) that might also induce ROS production and/or cell death.

There are few papers focused on the effects of ASK1 on myogenesis. We chose this target since ASK1 has been previously shown to be activated by oxidative stress and it is known to lie upstream of both the JNK and p38 MAPK apoptotic pathways [47]. It was felt that this target would give us the most information, and serve as a starting point for future studies between DUOXA1 and apoptosis. A recent investigation by Han and coworkers [48] suggests that, apart from initiating cell death, p38 MAPK and JNK activation enhance myostatin expression. Myostatin is a negative regulator of skeletal muscle mass [49,50]. Since ASK1 lies upstream of both p38 MAPK and JNK, it follows that its stimulation might enhance myostatin expression and result in decreased myocyte fusion. Clear links between H_2_O_2_ and myostatin expression remain to be established, but a recent investigation determined that C2C12 cells treated with myostatin produced higher levels of ROS than did controls [51]. Future studies might better determine the link between ROS, ASK1, myostatin and myogenesis.

Similarly, *notch* genes are also implicated in differentiation. Originally, our lab characterized DUOXA1 as a Numb-interacting protein. Drosophila NIP (dNIP) was observed to anchor Numb as a crescent to one side of the plasma membrane shortly before cell division, thus ensuring daughter cells to inherit different amounts of Numb and acquire distinct cell fates [7]. In an attempt to address the biological function of dNIP, we generated fly lines with either maternal deletion of *nip* or those expressing a UAS-*nip*-RNAi transgene. While *nip* deletion led to growth arrest and death at the 1^st^ larval instar, NIP-knockdown flies survived to adulthood. However, these flies exhibited defects in pre-adult development, displayed an inability to handle oxidative stress, and had a significantly reduced life span [52]. Intriguingly, these phenotypes could be fully rescued by ubiquitous expression of a *UAS-nip* or a *UAS-nip*^*NN/AA*^, the latter producing a NIP mutant bearing double Asn to Ala mutations, shown to be defective in Numb binding [7,52]. These results suggest that dNIP is essential for *Drosophila* development, but its in vivo function may not be related to Numb binding. We have also recently determined that mammalian DUOXA1 and Numb show differences in expression patterns in the developing brain, and that overexpression of DUOXA1 in P19 cells does not affect the regulation of Numb [22]. Thus, based on our recent findings in *Drosophila*, mouse brain and P19 cells, it is unlikely that interactions between DUOXA1 and Numb are functionally relevant.

## Conclusion

This is the first report of DUOXA1 in satellite cells and primary myoblasts, and the results of our work suggest this protein (as has been demonstrated in the thyroid and lung) is partially responsible for ROS production in developing muscle and that tight control of its levels is necessary for optimal myogenesis. Despite the presence of DUOXA1 and DUOX1 in these cells throughout muscle development, our work suggests that their levels need to be strictly controlled. As outlined in Figure 6, our work demonstrates that constitutive overexpression of DUOXA1 induces apoptosis and inhibits differentiation through mechanisms involving DUOX1 and ASK1. However, it remains possible that DUOX1-independent mechanisms also contributed to the phenotype associated with overexpression. DUOXA1 is localized in both the cytoplasm and nucleus in dividing myoblasts, while DUOX1 appears to be restricted to the plasma membrane. This result is consistent with previous observations in which DUOXA1 is associated with internal membranes, but remains crucial for the maturation and/or translocation of DUOX1 to the periphery of the cell [8]. The nuclear presence of DUOXA1 remains curious given its five transmembrane domains and well-documented association with DUOX1. Our lab has recently performed extensive mass spectrometry analysis to identify alternate binding partners for DUOXA1 in both the cytoplasm and nucleus [22]. Future investigations might seek to determine whether this protein has DUOX1-independent roles and whether it might be upregulated in diseased or aging muscle to determine its potential value as a therapeutic agent.

## Materials and methods

### Myofibre isolation and cell culture

Adult CD57/BL6 mice (> 6 weeks of age) were used for myofibre and primary myoblast isolations. Mice were housed and bred in the Health Sciences Animal Care Facility at the University of Western Ontario, and all procedures were monitored under a protocol approved by the University of Western Ontario Council on Animal Care. Mice were killed by cervical dislocation and myofibres were isolated as previously described [53]. Briefly, extensor digitorum longus (EDL) muscles were dissected from the hindlimbs and digested in collagenase D (Roche Applied Science, Mississauga, ON, Canada) for one hour at 37°C. Individual fibres were plated onto glass bottom dishes (MatTek corp, Ashland, MA) coated in 10% matrigel (BD Biosciences, San Jose, CA), and either fixed immediately in 2% paraformaldehyde (PFA) or cultured in plating medium for up to several days in Dulbecco’s Minimum Essential Medium (DMEM, Sigma, St. Louis, MO), 10% horse serum (HS, Gibco, Carlsbad, CA), 0.5% chick embryo extract (CEE, US Biologicals, Swampscott, MA) with streptomycin and penicillin (GIBCO) at 37°C in 5% CO_2_. In order to determine whether satellite cells had entered into the cell cycle, myofibres were labelled with 10 μM Bromo-deoxy Uridine (BrdU, Sigma) at the time of plating and harvested after 24 hours in culture.

In order to generate primary myoblast cultures, myofibers were washed from the plates after three days of culture and the medium was switched to growth medium (GM) containing DMEM, 10% HS, 20% fetal bovine serum (FBS, Biowest, Miami, FL), 1% CEE, and 2.5 ng/mL basic fibroblast growth factor (bFGF, Promega, Madison, WI). Myoblasts were maintained in this medium for up to several days. As cells reached 50-70% confluence, they were passaged after pre-plating for 15 minutes on matrigel coated dishes to remove fibroblasts, and plated on fresh matrigel coated dishes. The purity of the myoblast cultures was estimated by desmin staining to be > 95%. In order to maintain the characteristics of the cells, all experiments were carried out on myoblasts that had undergone 4 – 7 passages.

For experiments where cells were differentiated, cells were plated on matrigel coated dishes and grown until 50% confluent. At that time, GM was exchanged for differentiation medium (DM) containing DMEM, 2% HS, 10% FBS, 0.5% CEE, and antibiotics. Cells were differentiated for 48 hours unless otherwise stated, harvested and analysed. At the time of harvest, primary myoblasts were fixed in 2% PFA for 15 minutes and washed several times in phosphate buffered saline (PBS) and prepared for immunostaining.

### Adenoviral preparation

All adenoviral and corresponding control vectors were obtained from MP Biomedicals (Montreal, QC). Full-length mouse DUOXA1 was cloned into the BglII site of the CMV5-IRES-EGFP AdenoVator^TM^ vector to create CMV5-DUOXA1-IRES-EGFP and sequencing was performed. The final adenoviral vector was created by homologous recombination of the aforementioned vector with AdEasy, and virus was generated and amplified in 293T cells. Viral purification was achieved using an Adeno-X Virus Purification Kit (Clontech, Mountain View, CA). CMV5-IRES-EGFP containing virus was used as the corresponding control.

### Viral infection

Primary myoblasts were plated and maintained in growth medium until they reached 50-60% confluence. At this time, cultures were infected with either CMV5-DUOXA1-IRES-EGFP or CMV5-IRES-EGFP containing viruses. Twenty-four hours after infection, GM was replaced with DM, and cells were harvested after 48 hours of differentiation, unless otherwise stated. Samples were harvested for mRNA, analyzed by microscopy or prepared for H_2_O_2_ determination.

### shRNA-mediated knockdown of DUOXA1 and siRNA-mediated knockdown of DUOX1 or ASK1

Short hairpin RNA (shRNA) constructs targeting two separate regions of the DUOXA1 gene and a control construct targeting luciferase (3 μg) were used in knockdown experiments. All DUOXA1 shRNA constructs and controls were purchased from OriGene (Rockville, MD). At the appropriate cell density, myoblasts (6 × 10^5^ cells/cuvette) were electroporated using an Amaxa Nucleofector unit and NHDF solution (Lonza, Walkerville, MD). Twenty-four hours after nucleofection, GM was replaced with DM, and cells were harvested after 48 hours of differentiation. Samples were harvested for mRNA, analyzed by microscopy or prepared for H_2_O_2_ determination. In order to determine whether knocking down DUOX1 or ASK1 might rescue the phenotype corresponding to DUOXA1 overexpression, siRNA constructs targeting DUOX1, ASK1 or a scrambled control were purchased from Santa Cruz (Santa Cruz, CA). Small interfering RNA was introduced into proliferative primary myoblasts using nucleofection described above. Twenty four hours after nucleofection, samples were infected with adenoviral vectors containing GFP-DUOXA1 or GFP alone. Differentiation was initiated 24 hours after infection and samples were harvested 24 or 48 hours later. Sequences used in the preparation of siRNA and shRNA are presented in Additional file 2: Table S1.

### Immunostaining

Myofibres and myoblasts were permeabilized in 0.5% and 0.2% triton-X 100, respectively. After blocking for one hour in 1% bovine serum albumin (BSA), samples were incubated overnight at 4°C in a solution containing antibodies against MyoD (1:50), myogenin (1:50, Abcam, Cambridge, MA), BrdU (1:1500), myosin heavy chain (neat) (Developmental Studies Hybridoma Bank, Iowa), desmin (1:200, Molecular Probes, Eugene, OR), and DUOX1 (1:30, Santa Cruz, Santa Cruz, CA). For DUOXA1 detection, an antibody was generated in our lab against the C-terminal portion of the protein (CFKEEHPKESD) and validated against a blocking peptide [22]. Anti-DUOXA1 was used at a dilution of 1:300. Samples were washed and visualized with Alexa-Fluor secondary antibodies diluted 1:1000 (Molecular Probes). 4′6–diamidino-2-phenylindole (DAPI, Sigma) was used as a nuclear marker. Confocal microscopy was performed on a Zeiss LSM 510 META confocal microscope (Carl Zeiss, Germany) using 20×, 40× or 63× objectives. Images were collected using Laser Scanning Microscope (LSM) software and optimized using PhotoImpression5 software.

### Apoptosis assays

Apoptotic cells were identified using an Annexin V-Cy3 kit according to the manufacturer’s instructions (BioVision, Mountain View, CA). Briefly, live cells were incubated in binding buffer supplied with the kit, along with Annexin V-Cy3 (1:100), propridium iodide (TOPRO-3, 1:1000) and Hoechst 33342 (1:2000, Molecular Probes). Samples were maintained in a heating block set to 37°C during analysis, and cells undergoing early (Annexin V^+^/TOPRO-3^-^) or late (Annexin V^+^/TOPRO-3^+^) apoptosis were compared with the total number of cells (identified with Hoechst dye). In overexpression experiments where GFP could be used as a marker, only GFP^+^ cells were included in the analysis. Hoechst dye, GFP, Annexin V-Cy3 and TOPRO-3 were visualized using a Zeiss LSM 510 META confocal microscope, with excitation lasers set to 405 nm, 488 nm, 543 nm and 633 nm, respectively.

### RNA extraction, cDNA synthesis and quantitative reverse transcription (qRT)-PCR

Total RNA was extracted from samples using TRIzol reagent, according to the manufacturer’s instructions (Invitrogen, Burlington, ON). First-strand cDNA was generated from 100 to 300 ng RNA using the QuantiTect Reverse Transcription kit (Qiagen, Toronto, ON), which provides an initial step to eliminate genomic DNA. The samples were diluted and 1/15 of this mixture was quantified in subsequent PCR reactions using PerfeCTa SYBR Green SuperMix (Quanta Biosciences, Gaithersburg, MD). Samples were analyzed using the Rotor-Gene Q (Qiagen, Toronto, ON) and the corresponding software. Relative gene expression was calculated using the Ct method, and all samples were normalized to glyceraldyhyde-3-phosphate dehydrogenase (GAPDH). All averages ± S.D. are displayed as fold changes relative to gene levels at d0 or to GFP control cells, depending on the experiment. Primer pairs were derived from the PrimerBank ( http://pga.mgh.harvard.edu/primerbank/), or from previous publications, and are listed in Additional file 3: Table S2.

### Measurement of H_2_O_2_ using Amplex Red

Hydrogen peroxide production was determined using an Amplex Red kit (Molecular Probes), according to the manufacturer’s instructions. In the presence of peroxidase, Amplex Red reagent reacts with H_2_O_2_ (in 1:1 stoichiometry) to produce a red fluorescent product called resoruffin. The high extinction coefficient of resoruffin allows for analysis either fluorometrically or spectrophotometrically. Aliquots of medium were subsequently removed and analyzed spectrophotometrically at a wavelength of 560 nm. After H_2_O_2_ determination, samples were washed thoroughly and corrected for cell number using a CytoSelect colormetric assay kit (Cell Biolabs, Inc., San Diego, CA). Dye from the stained cells was extracted and quantified at OD 560 nm.

### Statistical analysis

Where primary myoblasts were quantified by microscopy for a given antigen, cells from at least 10 random fields were counted and scored. Primary myoblasts from at least three mice were analysed. Images were optimized and assembled into figures using Adobe Illustrator. In order to determine the fusion index, the number of structures containing 2 or more nuclei were analysed from at least three separate mice. The fusion index was calculated as:

$$\begin{array}{l} \left(\#\;\text{of fused cells containing at least}2\;\text{nuclei}/\text{total}\;\#\right)\\ \left(\text{of cells}\right)x100 \end{array}$$

In overexpression experiments (where GFP was available as a marker), GFP^+^ cells were counted for quantification and fusion was calculated as:

$$\begin{array}{l} \left(\#\;\text{of fused}{\text{GFP}}^{+}\text{cells containing at least}2\;\text{nuclei}/\right)\\ \;\left(\text{total}\#\;\text{of}{\text{GFP}}^{+}\text{cells}\right)x100 \end{array}$$

P < 0.05 was considered significantly different between conditions, and was calculated using a Student’s *t*-test.

## Abbreviations

NIP: Numb interacting protein; DUOX: Dual oxidase; DUOXA1: Dual oxidase maturation factor 1; ROS: Reactive oxygen species; ASK1: Apoptosis signal-regulating kinase 1; BrdU: Bromo-deoxy uridine; GM: Growth medium; DM: Differentiation medium; shRNA: Short hairpin RNA; siRNA: small interfering RNA; qRT: quantitative reverse transcription.

## Competing interests

The authors declare that they have no competing interests.

## Authors’ contributions

SS designed, performed and analyzed experiments and wrote the manuscript. KK analyzed RNA samples. XX designed the peptide which was used to produce the DUOXA1 primary antibody. JGP contributed to the gene transfer studies and helped draft the manuscript. SL supervised the experiments and helped draft the manuscript. All authors read and approved the final manuscript.

## Supplementary Material

Additional file 1: Figure S1 (A) In order to determine the specificity of a lab-derived anti-DUOXA1 antibody, Western blotting was performed on 293 T cell samples transfected with either DUOXA1 or the corresponding empty vector (CON). (B) Since DUOXA1 is known to be glycosylated [16], we subjected 293 T cell lysates overexpressing DUOXA1 to N-glycosidase-F for 2 hr at 37°C. Results demonstrate that deglycosylation results in the DUOXA1 band migrating to its predicted weight of 37 kDa. (C-D) Immunostaining was on primary mouse myoblasts. Samples were incubated overnight with a rabbit anti-DUOXA1 antibody either in the absence (C), or presence (D) of a blocking peptide (BP), and visualized with an Alexa 488 conjugated secondary antibody. Scale bars: 50 μm. Inset scale bars: 10 μm. (E) Further support was derived from adenoviral infection of the immortalized C2C12 myoblast cell line. Cells successfully infected with GFP-DUOXA1 show bright green fluorescence (green) along with bright DUOXA1 staining (red) indicating substantial overexpression of DUOXA1. GFP control cells do not demonstrate elevated levels of DUOXA1. Scale bars: 20 μm. Click here for file

Additional file 2: Table S1 Sequences used for siRNA and shRNA construction. Click here for file

Additional file 3: Table S2 Primers used for qRT-PCR. Click here for file
