# Supplementary figures and images for: Dual Oxidase Maturation factor 1 (DUOXA1) overexpression increases reactive oxygen species production and inhibits murine muscle satellite cell differentiation
Source: Cell Commun Signal. 2014 Jan 11;12:5. doi: 10.1186/1478-811X-12-5 (PMC3895674; doi:10.1186/1478-811X-12-5)

Supplemental Figure 1S

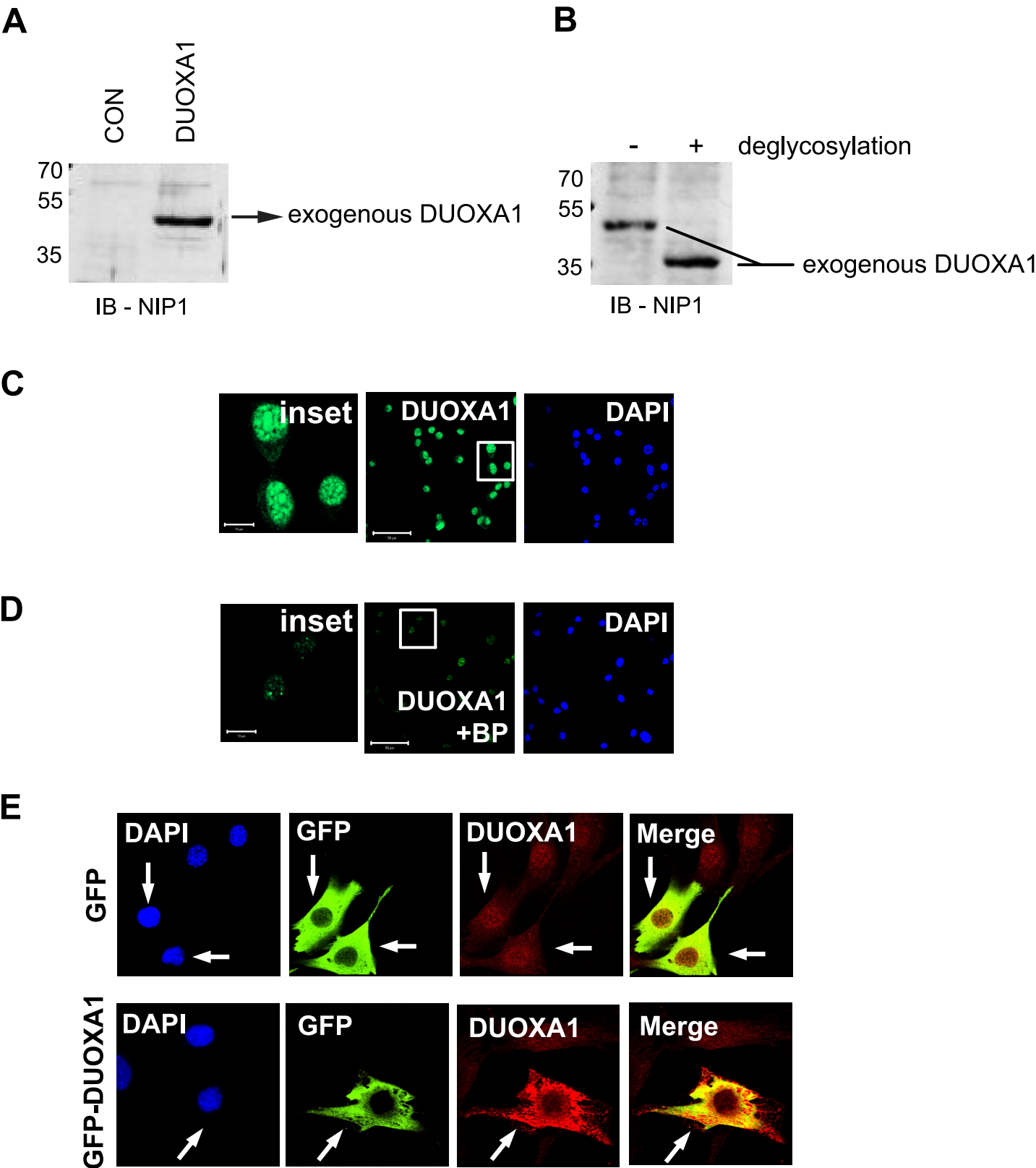

Supplement: Additional file 1: Figure S1 — (A) In order to determine the specificity of a lab-derived anti-DUOXA1 antibody, Western blotting was performed on 293 T cell samples transfected with either DUOXA1 or the corresponding empty vector (CON). (B) Since DUOXA1 is known to be glycosylated [16], we subjected 293 T cell lysates overexpressing DUOXA1 to N-glycosidase-F for 2 hr at 37°C. Results demonstrate that deglycosylation results in the DUOXA1 band migrating to its predicted weight of 37 kDa. (C-D) Immunostaining was on primary mouse myoblasts. Samples were incubated overnight with a rabbit anti-DUOXA1 antibody either in the absence (C), or presence (D) of a blocking peptide (BP), and visualized with an Alexa 488 conjugated secondary antibody. Scale bars: 50 μm. Inset scale bars: 10 μm. (E) Further support was derived from adenoviral infection of the immortalized C2C12 myoblast cell line. Cells successfully infected with GFP-DUOXA1 show bright green fluorescence (green) along with bright DUOXA1 staining (red) indicating substantial overexpression of DUOXA1. GFP control cells do not demonstrate elevated levels of DUOXA1. Scale bars: 20 μm. [file 1478-811X-12-5-S1.pdf]
