# Supplementary material for: Dual Oxidase Maturation factor 1 (DUOXA1) overexpression increases reactive oxygen species production and inhibits murine muscle satellite cell differentiation
Source: Cell Commun Signal. 2014 Jan 11;12:5. doi: 10.1186/1478-811X-12-5 (PMC3895674; doi:10.1186/1478-811X-12-5)
Supplement: Additional file 2: Table S1 — Sequences used for siRNA and shRNA construction. [file 1478-811X-12-5-S2.doc]

*Table S1: Sequences used for shRNA and siRNA*

**DUOXA1 shRNA (Origene) sequences used to silence DUOXA1:**

**Product GI592486:** TACTCTGGCTGTCATTATCACCATCTTCC

**Product GI592489:** ATGTTGTCGATGCCAGTGCTGGTTTATGG

**DUOX1 and ASK1si RNA (Santa Cruz Biotechnology) used to silence DUOX1 and ASK1:**

**The sequence of sc-37007 - Control siRNA-A is:**

• Sense: UUCUCCGAACGUGUCACGUTT

• Antisense: ACGUGACACGUUCGGAGAATT

**sc-29749 : ASK 1 siRNA (m) is a pool of 3 different siRNA duplexes:**

**sc-29749A:**

• Sense: CAGCAGACAUUGUUAUCAATT

• Antisense: UUGAUAACAAUGUCUGCUGTT

**sc-29749B:**

• Sense: CUUCUUACCUGUCUAUCAATT

• Antisense: UUGAUAGACAGGUAAGAAGTT

**sc-29749C:**

• Sense: CCUGAACUUUGAAGUAGAATT

• Antisense: UUCUACUUCAAAGUUCAGGTT

**sc-60551 : DUOX1 siRNA (m) is a pool of 3 different siRNA duplexes:**

**sc-60551A:**

• Sense: GGAACGGAUUGUUCUCUAATT

• Antisense: UUAGAGAACAAUCCGUUCCTT

**sc-60551B:**

• Sense: CUGGUUCUGUUGUUUAACATT

• Antisense: UGUUAAACAACAGAACCAGTT

**sc-60551C:**

• Sense: GAAGGAGCACUGUUUAAGATT

• Antisense: UCUUAAACAGUGCUCCUUCTT
