# Supplementary material for: Dual Oxidase Maturation factor 1 (DUOXA1) overexpression increases reactive oxygen species production and inhibits murine muscle satellite cell differentiation
Source: Cell Commun Signal. 2014 Jan 11;12:5. doi: 10.1186/1478-811X-12-5 (PMC3895674; doi:10.1186/1478-811X-12-5)
Supplement: Additional file 3: Table S2 — Primers used for qRT-PCR. [file 1478-811X-12-5-S3.doc]

*Table S2: Primer list*

| **GENE** | **Accession #** | **Forward Primer** | **Reverse Primer** |
| --- | --- | --- | --- |
| DUOXA1 | NM_145395.2 | TGCTGCTGGCCACTGGTCTCTTCC | CACTGACATGGGAGTGTCCTGGGTC |
| DUOX1 | NM_001099297.1 | ACCAGAACATTGCGATGTATGAG | AGAAATGGACGGTATCCTGGA |
| ASK1 | AB006787.3 | TGCTCACAGCGATGCCAAAG | GAAGCTACTGCAGGAGGGTA |
| MyoD | NM_010866.2 | CCCCGGCGGCAGAATGGCTACG | GGTCTGGGTTCCCTGTTCTGTT |
| Myf5 | NM_008656.5 | CCTGTCTGGTCCCGAAAGAAC | GACGTGATCCGATCCACAATG |
| Myogenin | NM_031189.2 | GCAATGCACTGGAGTTCG | ACGATGGACGTAAGGGAGTG |
| MyHC | NM_030679.1 | ACAACCCCTACGATTATGCGT | ACGTCAAAGGCACTATCCGTG |
| GAPDH |  | CAT GGC CTT CCG TGT TCC TA | CCT GCT TCA CCA CCT TCT TGA |
